# Empowering learning in crystallography: launch of the *Education and outreach* section in *Acta Crystallographica* Section E

**DOI:** 10.1107/S2056989025005109

**Published:** 2025-09-05

**Authors:** Shao-Liang Zheng, Sean Parkin

**Affiliations:** a Department of Chemistry and Chemical Biology Harvard University 12 Oxford St Bauer B06 Cambridge MA 02138 USA; b Department of Chemistry University of Kentucky Lexington KY 40506-0055 USA

**Keywords:** crystallography education, elementary/secondary school outreach, undergraduate/graduate education, undergraduate/graduate research

## Abstract

This editorial announces the launch of the *Education and outreach* section in *Acta Crystallographica Section E*, inspired by the success of the 2023 virtual issue on *Modern approaches and tools for teaching crystallography*. The section invites detailed advice on challenges encountered during structure solution and refinement, as well as tested educational activities, outreach reports, and practical teaching resources in crystallography and structural chemistry, with an emphasis on reproducibility and student involvement. Inaugural contributions are highlighted, and global submissions are welcomed to build a collaborative resource for advancing education in structural science.

## Abstract 2

The *Acta Crystallographica Section E* announces a call for papers for its new *Education and outreach* section, dedicated to showcasing innovative practices, resources, and insights in crystallography and structural chemistry education and public engagement.

Crystallography and structural chemistry flourish through a vibrant culture of discovery, teaching, and community engagement. Across the globe, learners and educators – from elementary and secondary school students to established researchers– are drawn to our field by the wonder of visualizing and understanding the structure of matter. As techniques and technologies advance, education and outreach in structural science must keep pace, preparing our next generation to meet the challenge and adapting our approaches to sharing knowledge and inspiring participation.

With this vision, *Acta Crystallographica Section E* is delighted to announce the launch of the *Education and outreach* section, a dedicated platform to showcase, support, and inspire innovative educational practices and public engagement in crystallography around the world.

This new section is directly inspired by the enthusiastic response to the 2023 special virtual issue *Modern approaches and tools for teaching crystallography* (Díaz de Delgado & Parkin, 2023 ▸). That collection brought together creative didactic methods and practical tools, highlighting the depth and breadth of talent within our worldwide community. The positive energy generated by that issue has convinced us to provide an ongoing home for the discussion and dissemination of educational and outreach practices in crystallography and structural chemistry.

The new *Education and outreach* section welcomes articles encompassing all aspects of crystallography education and outreach related to structural chemistry and the broader field of structural science. Alongside detailed advice on addressing challenges during structure solution and refinement, such as symmetry determination (Clegg, 2019 ▸), disorder modelling (Parkin *et al.*, 2023 ▸; Vinaya *et al.*, 2023 ▸), twinning handling (Parkin, 2021 ▸), and validation (Linden, 2020 ▸; Spek, 2020 ▸), we especially encourage:

**·Tested hands-on activities and learning modules**: Whether developed for elementary, secondary, or university classrooms, labs, or informal settings such as museums and public demonstrations, we encourage practical, engaging activities that have been implemented and evaluated with students. Activities should introduce thought-provoking topics, spark discussion, and be based on authentic teaching or outreach experience, with reflection and evidence of success, not simply proposed ideas (Wouters & Van Meervelt, 2022 ▸; Meurer *et al.*, 2024 ▸; Zheng *et al.*, 2025 ▸).

**·Outreach and capacity building reports**: Share your journeys in broadening access to crystallography, introducing the subject in schools, building science capacity in under-resourced communities, or running community events, from science festivals to crystal-growing competitions (Orton *et al.*, 2023 ▸; Billinge, 2024 ▸; Kenfack Tsobnang *et al.*, 2024 ▸; Bou-Nader *et al.*, 2025 ▸; Murray *et al.*, 2024 ▸).

**·Teaching insights, tips, and best practices:** Distil your ‘tips and tricks’, effective protocols, and instructional materials to empower fellow educators and make crystallography more accessible and inspiring for learners at all levels (Foxman, 2021 ▸; Zheng & Campbell, 2021 ▸; Massera & Helliwell, 2023 ▸; de la Flor *et al.*, 2024 ▸).

To empower educators and outreach organizers worldwide, we encourage detailed supporting information, such as instructor notes including sources for materials, ready-to-use student handouts, implementation guidance, and demonstration videos where available. These resources will make it easier for others to adapt activities, extend their reach, enhance reproducibility, and maximize educational impact.

We particularly welcome submissions showcasing undergraduate-led structure determinations and course-based projects (Stiers *et al.*, 2016 ▸; Orton *et al.*, 2023 ▸), where authentic student engagement and discovery are foregrounded. Undergraduate authorship is encouraged when students take a leading role in writing and sharing their work, and a special ‘student project’ note may be included for an extensive author list. Interdisciplinary projects with a chemical education component are also welcome, provided that crystallographic content remains central (Orton *et al.*, 2023 ▸).

With this new section, we aim to strengthen the bonds across our worldwide crystallography community. We wel­come both seasoned educators and first-time teachers, experienced researchers, outreach leaders, and curious students to contribute. Expanded versions of presentations from Education sessions at IUCr Congresses (Gražulis *et al.*, 2015 ▸; Flor *et al.*, 2024 ▸; Bucio *et al.*, 2024 ▸; Meurer *et al.*, 2024 ▸; Murray *et al.*, 2024 ▸; Zheng *et al.*, 2025 ▸) or the local meetings organized by ACA (Clegg, 2019 ▸; Bou-Nader *et al.*, 2025 ▸), AfCA, AsCA, ECA (Clegg, 2019 ▸; Linden, 2020 ▸), and LACA, materials from crystallography schools and workshops, and especially reports on IUCr outreach initiatives are particularly encouraged.

We are privileged to kick off this new section with contributions from leading voices in our field:

**·** Christina Hoffmann and Xiaoping Wang will describe educational and research applications of modern neutron Laue time-of-flight methods in structural science.

**·** Graciela Diaz de Delgado and collaborators will present a step-by-step account of the structure determination of a pharmaceutical from powder diffraction data.

**·** Dilek Kiper Dogutan and colleagues (Dogutan *et al.*, 2025 ▸) present a new instructional module designed to inspire undergraduates in chemistry and structural science.

**·** Sean Parkin (2025 ▸) shares a strategy for handling unusual weighting schemes sometimes encountered in structure refinements with *SHELXL*.

**·** Joseph Reibenspies will share practical ‘tips and tricks’ in structural science drawn from his distinguished career.

**·** Stacey Smith and collaborators will share practical suggestions for implementing powder diffraction using a single-crystal diffractometer.

**·** Richard Staples (2025 ▸) describes crystal growth for X-ray diffraction, a cornerstone of hands-on learning in structural chemistry.

**·** Yu-Sheng Chen and collaborators will showcase advanced crystallography applications on dedicated single-crystal synchrotron beamlines.

These inaugural contributions, complemented by the other exemplary works we have referenced, set the tone for the diverse, impactful content we aim to feature and highlight the value of sharing experiences across the educational spectrum.

On behalf of the editorial team, we look forward to your submissions and your insights as we grow this new section together.

*Let us shape the future of crystallography – through education, outreach, and community.*
